# 

**Published:** 1887-01

**Authors:** 


### List of Original Contributors to Vol. VIII.

| | |
|---|---|
| BARRETT, W. C | M. D., D. D. S., M. D. S. |
| BEERS, W. GEO | L. D. S. |
| BLACK, G. V | M. D., D. D. S. |
| BODECKER, C. F. W | D. D. S., M. D. S. |
| BRIMMER, F. H | D. D. S. |
| BRYAN, L. C | D. D. S. |
| CURTISS, G. L | M. D., D. D. S. |
| DARBY, F. B | D. D. S. |
| FARRAR, J. N | M. D., D. D. S. |
| FILLEBROWN, THOMAS | M. D., D. M. D. |
| GERAN, J. P | DR. |
| HARLAN, A. W | M. D.. D. D. S. |
| HASKELL, L. P | DR. |
| HEITZMANN, CARL | M. D. |
| HOPKINSON, B. MERRILL | D. D. S., M. D. |
| IVY, ROBERT S | D. D. S. |
| JENKINS, N. S | D. D. S. |
| JOHNSON, C. N | L. D. S., D. D. S. |
| KING, HERBERT M | M. D. |
| KINGSLEY, N. W | D. D. S, |
| KOCH, C. R. E | D. D. S. |
| LAND, C. H | DR. |
| LEWIS, GEO. W., JR | A. M., M. D. |
| LOW, F. W | DR. |
| MARVIN, C. A | D. D. S. |
| MAXFIELD, GEO. A | D. D. S. |
| MAYR, CHAS | A. M. |
| MILLER, W. D | A. B., PH.D., D. D. S. |
| MILLS, GEO. A | DR. |
| MITCHELL, W. H | D. D S. |
| MOODY, J. D | D. D. S. |
| PALMER, S. B | M. D. S. |
| PHILLIPS, C. E. H | D. D. S. |
| RISHEL, GEO. P | D. D. S. |
| ROLLINS, W. H | M. D. |
| TERRY, C. T | D. D. S. |
| TRUEMAN, W. H | D. D. S. |
| WEAGANT, GEO. H | L. D. S. |

### INDEX.

TO THE

### INDEPENDENT PRACTITIONER.

### VOL. VIII.

### ORIGINAL COMMUNICATIONS.

**A**bsorption of Dentine; Its Relation to the Process of Reimplantation and to Decay of the Teeth. Prof. Miller ........ 16
Alveolar Abscess. Geo. A. Maxfield ........ 349
A Bold Declaration Reviewed. C. A. Marvin ........ 57

**C**auses and Treatment of Dental Caries. Geo. P. Rishel ........ 631
Clinic of Prof. Garretson. Robert S. Ivy ........ 298, 411
Concerning Some Uses of Oxy-Chloride of Zinc. N. S. Jenkins ........ 123
Contributions to the History of Development of the Teeth. Carl Heitzmann and C. F. W. Bödecker ........ 225, 281, 337, 397, 453, 509, 565, 621
Copper Amalgam. Geo. H. Weagant ........ 625
Courtesy Among Dentists at Professional Meetings. J. N. Farrar ........ 71

**D**entistry and Medicine. Herbert M. King ........ 90
Dentistry Not a Specialty in Medicine. N. W. Kingsley ........ 1
Discriminate Use of Gold. S. B. Palmer ........ 175
Dividing Line, The. Frank W. Low ........ 406
Dr. Younger and Implantation of Teeth. C. A. Marvin ........ 25
Driving Wheels and Strokes in a Dental Engine. W. H. Rollins ........ 248

**E**lements of Success, The. W. C. Barrett ........ 235

**G**eneral Health as Expressed by the Work of the Kidneys, and its Relation to the Teeth. Chas. Mayr ........ 578

**H**ard Rubber and Corundum Disks and Wheels. Geo. A. Maxfield ........ 520
Herbst Fillings. J. P. Geran ........ 19
Herbst Method of Filling Teeth, The. C. F. W. Bödecker ........ 78

**I**mplantation of Teeth. G. L. Curtiss ........ 21
Influence of Culture on Professional Skill, The. Thos. Fillebrown ........ 113
Is This True? C. R. E. Koch ........ 126

**M**etallic Enamel Sections. A New System for Filling Teeth. Dr. C. H. Land .......... 87
Metallic Enamel Coatings and Fillings. C. H. Land .......... 413
My Student. J. D. Moody .......... 518
Metal Work. L. P. Haskell .......... 133

**O**ral Hygiene and Care of the Teeth. C. E. H. Phillips .......... 187
Ovarian Cyst, An. W. C. Barrett .......... 294
Overcoming Leverage in Superior Dentures. L. C. Bryan .......... 192

**P**ain Obtunder, A. F. H. Brimmer .......... 301
Post-Graduate Study. C. N. Johnson .......... 189
Prehistoric Decay of the Teeth. C. T. Terry .......... 27

**R**ecent Theories of the Formation of Pus, Considered with Reference to Dental Operations. G. V. Black .......... 462
Robert Koch—His Work and Methods. Geo. W. Lewis, Jr .......... 290

**S**ome Observations from the Life of the Late Dr. John M. Riggs, of Hartford, Conn. Geo. A. Mills .......... 129
Stenocarpin. Wm. H. Mitchell .......... 523
Surgical and Therapeutic Treatment of Caries and Necrosis of the Alveolar Process in Man, The. A. W Harlan .......... 169

**T**eeth with Exposed Pulps. B. Merrill Hopkinson .......... 184
To Be or Not To Be. J. G. Palmer .......... 256
Tooth-Pulp in its Relations to Dental Operations, The. Wm. H. Trueman. 569
Two Dentists who do "Not Get On." W. Geo. Beers .......... 635

**V**isit to Foreign Dental Schools and Other Observations, A. A. W. Harlan 402

"**W**anted—A First-Class Mechanical Dentist. No Student or Operator Need Apply." Frank B. Darby .......... 181

### REPORTS OF SOCIETY MEETINGS.

**A**merican Dental Association .......... 480, 525, 582, 638
American Dental Society of Europe .......... 654

**C**entral Dental Association of Northern New Jersey .......... 194, 249, 318, 597
Clinic of the Chicago Dental Club .......... 312
Connecticut Valley Dental Society .......... 492, 536, 596, 650

**D**ental Society of the State of New York, The .......... 310

**F**irst District Dental Society of New York .......... 35, 93, 135

**I**llinois State Dental Society .......... 303, 363, 415

**M**innesota State Dental Society .......... 429

**N**ational Association of Dental Examiners .......... 487
New Jersey State Dental Society .......... 547
New York Odontological Society .......... 30, 96, 260, 315, 599, 659
Ninth International Medical Congress, Washington, D. C. .......... 528, 588, 641

**S**outhern Dental Association .......... 541, 592, 647

### EDITORIAL.

Action of the American Medical Association, The Late ........ 350
A. D. A. Meeting for 1887, The ........ 320, 377, 500
American Dental Association ........ 211, 265
American System of Dentistry ........ 162
Anniversary Meeting ........ 45
Association, The ........ 435
At Washington ........ 556

Bleaching Teeth ........ 608
Bonwill Dental Engine and Mallet, The ........ 330
Brethren Across the Line, Our ........ 553
Broken Broaches in Pulp Canals ........ 103

Can There be Suppuration without the Presence of Micro-Organisms? ........ 104
Concerning Contests ........ 496
Complimentary ........ 162
Congratulatory ........ 555
Congress, The ........ 550
Crowded Out ........ 162

Dental Societies in America ........ 106
Dentifrices ........ 206

Editorially ........ 664
Educational Matters ........ 663

First District Dental Society Meeting and Some of its Results, The ........ 99

Immediate Root-Filling ........ 605
Implantation ........ 43, 385
In Extenuation ........ 501
It Can Wait ........ 501

"Let Us Have Peace" ........ 270

Medical Congress, The ........ 155, 498
Medical Recognition ........ 430
Medical Recognition Again ........ 602
Medicine and Dentistry ........ 41

New Remedies ........ 433
New York Society Reports ........ 158

Personal ........ 45
Practical Scheme, A ........ 328
Professional Courtesy ........ 160
Prospective ........ 210
Pus Formation ........ 501

Receipts ........ 45
Remittances ........ 211
Rest for Dentists ........ 325

Society Objects.................................................. 556
Subscribers, To.................................................. 105

That Two Hundred Dollar Prize.................................... 499
To Junior Dentists............................................... 501
Tooth-Crown Patents, The......................................... 208
Tooth Powders.................................................... 434

Unusual Opportunity, An.......................................... 606

Vale! 1887....................................................... 662

Waite Testimonial Fund, The...................................... 267
Washington Meeting, The.......................................... 384
Western Dental Journal, The...................................... 105

### BIBLIOGRAPHICAL.

..........................................45, 212, 386, 435, 557, 560, 609

### CURRENT NEWS AND OPINION.

..........................49, 107, 163, 216, 271, 331, 386, 442, 502, 560, 616, 665

### PUBLISHED BY DENTISTS FOR DENTISTS.

### Prospectus.

THIS JOURNAL is published by an association of professional men who have no other object than to afford to the profession an independent, outspoken Journal, which shall be the exponent of higher aims and a better practice.

Having no connection with any school, clique, or advertising firm, it will be impartial in its judgment of professional matters, and its appeals for support are directed to those who believe that a liberal profession should have an independent literature.

While not neglecting practical details, it will pay especial attention to those oral diseases that have usually been under the care of the general practitioner, but which more properly belong to the specialty of dentistry.

Its pages will be open to any member of the profession who has anything of interest to communicate, and it invites a free expression of views on all subjects, medical or dental.

As each of the gentlemen forming the association is practically a member of the editorial corps, it will have abundant opportunity for collecting all the latest professional news and intelligence.

It will have a body of regular contributors, selected from the very best writers in the profession, and will thus be certain of an abundance of interesting original matter.

It will pay especial attention to reports of the meetings of dental societies, and its aim will be to publish an epitome of all important discussions of professional matters.

It will contain original abstracts and translations of all that is of moment in the foreign journals.

Its profits, if any, will be devoted to its improvement by adding needed illustrations, paying for valuable original communications, and enlarging its size whenever this becomes advisable.

To the accomplishment of the work thus outlined (necessarily relying upon the hearty approval of an appreciative profession), each of the publishers of the INDEPENDENT PRACTITIONER has pledged his best efforts.

Editorial communications, *and everything relating to the subscription department*, should be addressed to DR. W. C. BARRETT, No. 208 Franklin St., Buffalo, N. Y. Send all other business letters to DR. WILLIAM CARR, 35 West Forty-Sixth Street, New York.

| | | |
|---|---|---|
| ABBOTT, FRANK..N. Y. | CARR, WILLIAM .....New York. | HILL, O. E......Brooklyn. |
| BARRETT, W. C.Buffalo. | DUDLEY, A. M......Salem, Mass. | MILLER, W. D.Berlin,Ger. |
| BODECKER, C.F.W.N.Y. | FRANCIS, C. E....... New York. | PALMER, S. B...Syracuse. |

